# MIG-6 suppresses endometrial epithelial cell proliferation by inhibiting phospho-AKT

**DOI:** 10.1186/s12885-018-4502-7

**Published:** 2018-05-29

**Authors:** Jung-Yoon Yoo, Hee-Bum Kang, Russell R. Broaddus, John I. Risinger, Kyung-Chul Choi, Tae Hoon Kim

**Affiliations:** 1 Department of Obstetrics, Gynecology and Reproductive Biology, College of Human Medicine, Michigan State University, Grand Rapids, MI 49503 USA; 2 Department of Biochemistry and Molecular Biology, Yonsei University College of Medicine, Seoul, 03722 South Korea; 3 Department of Biomedical Sciences, ASAN Medical Center, University of Ulsan College of Medicine, Seoul, 05505 South Korea; 4 Department of Pathology, University of Texas M.D. Anderson Cancer Center, Houston, Texas TX 77030 USA; 5 Department of Pharmacology, Asan Institute for Life Sciences, Asan Medical Center, University of Ulsan College of Medicine, Seoul, 05505 South Korea

**Keywords:** MIG-6, Progesterone resistance, Endometrial hyperplasia, AKT

## Abstract

**Background:**

Aberrant hyperactivation of epithelial proliferation, AKT signaling, and association with unopposed estrogen (E2) exposure is the most common endometrial cancer dysfunction. In the normal uterus, progesterone (P4) inhibits proliferation by coordinating stromal-epithelial cross-talk, which we previously showed is mediated by the function of Mitogen-inducible gene 6 (*Mig-6*). Despite their attractive characteristics, non-surgical conservative therapies based on progesterone alone have not been universally successful. One barrier to this success has been the lack of understanding of the P4 effect on endometrial cells.

**Method:**

To further understand the role of *Mig-6* and P4 in controlling uterine proliferation, we developed a *Sprr2f-cre* driven mouse model where *Mig-6* is specifically ablated only in the epithelial cells of the uterus (*Sprr2f*^*cre+*^*Mig-6*^*f/f*^). We examined P4 effect and regulation of AKT signaling in the endometrium of mutant mice.

**Results:**

*Sprr2f*^*cre+*^*Mig-6*^*f/f*^ mice developed endometrial hyperplasia. P4 treatment abated the development of endometrial hyperplasia and restored morphological and histological characteristics of the uterus. P4 treatment reduced cell proliferation which was accompanied by decreased AKT signaling and the restoration of stromal PGR and ESR1 expression. Furthermore, our in vitro studies revealed an inhibitory effect of MIG-6 on AKT phosphorylation as well as MIG-6 and AKT protein interactions.

**Conclusions:**

These data suggest that endometrial epithelial cell proliferation is regulated by P4 mediated *Mig-6* inhibition of AKT phosphorylation, uncovering new mechanisms of P4 action. This information may help guide more effective non-surgical interventions in the future.

**Electronic supplementary material:**

The online version of this article (10.1186/s12885-018-4502-7) contains supplementary material, which is available to authorized users.

## Background

Endometrial cancer is the most common gynecologic malignancy in the United States, and in the last several decades the incidence of new cases each year has increased [1]. Endometrioid endometrial cancer, the most common type of endometrial cancer (80–85%), is associated with or preceded by abnormal multiplication of endometrial epithelial cells, known as complex atypical hyperplasia [2–4]. The main treatment for endometrial cancer is hysterectomy [5, 6]. Although most endometrial cancer diagnoses are in post-menopausal women, 5% of cases are diagnosed before age 40 and 20~ 25% before menopause [7]. Moreover, the incidence of endometrial cancer diagnoses in younger patients is likely to increase going forward due to increases in obesity, hypertension, diabetes mellitus, and other known endometrial cancer risk factors [8–10]. Therefore, the demand for non-surgical approaches to endometrial cancer is increasing, especially for women of reproductive age with complex atypical hyperplasia and early-stage endometrioid endometrial cancer who wish to preserve their fertility beyond treatment [8, 10].

Although hysterectomy is a key therapy for endometrial cancer [5, 6], recent intrauterine progestin therapies such as a levonorgestrel-releasing intrauterine system have been used for reproductive-aged women with complex atypical hyperplasia and early-stage endometrial cancer in cases when there is a desire to preserve fertility or when comorbidities exclude the possibility of surgery. In addition, progestin therapy is also considered for recurrent endometrial cancer because it is less toxic than chemotherapies; however, though the response rate of endometrial hyperplasia to progestin treatment is higher than that of endometrial adenocarcinoma, the response to progestin in cancer recurrence is worst of all. Progestin therapies used in the clinic are effective for some patients but not all cases of endometrial hyperplasia and well-differentiated endometrioid endometrial cancer. Another major limitation of progestin therapy is the lack of a clinical standard protocol for the type, dose, and duration of treatment [11–13]. The molecular mechanisms underlying progesterone (P4) resistance in endometrial cancer have not been fully understood.

Loss of control over uterine epithelial cell proliferation and apoptosis by ovarian steroid hormones is the major underlying pathogenesis of endometrial cancer [14–17]. Progesterone therapy can prevent this process by blocking actions of unopposed estrogen (E2) [18]. Nonetheless, several studies indicate that P4 therapy has low and unpredictable response rates in women with endometrial cancer, therefore limiting its potential use [19–23]. Resistance to P4 treatment due to loss of either progesterone receptor (PGR) itself or its signaling pathways causes significant difficulty in the treatment of advanced and recurrent endometrial cancer [24]. Identifying molecular mechanisms involved in P4 resistance is critical to effective and personalized treatment. Unfortunately, further translational research of endometrial cancer is inhibited by the lack of sufficient pre-clinical animal models.

Sequencing analysis of endometrial cancers in the Cancer Genome Atlas has revealed that upwards of 90% of cases of endometrioid endometrial cancer have some genetic aberration in the PTEN/PI3K pathway, which results in increased AKT activity [25]. In addition, the AKT signaling pathway can be activated by E2 [26] enhancing cell proliferation [27]. Therefore, an understanding of the molecular mechanisms between steroid hormone and PTEN/PI3K/AKT signaling will allow us to be in a much better position to develop new conservative therapies based on P4 function.

The protein structure of AKT consists of a PH domain, a linker region, a kinase domain, and a regulator domain [28]. These domains undergo various protein modifications including phosphorylation, acetylation, ubiquitylation, methylation, hydroxylation, glycosylation, and SUMOylation which help regulate the proteins activity [29]. AKT regulates different pathways that aid in the promotion of cellular survival and inhibition of apoptosis through its serine/threonine kinase activity [30, 31]. Inappropriately elevated expression of AKT phosphorylation is related to poor prognosis of endometrial cancer patients [32]. Furthermore, inhibition of the AKT pathway combined with P4 decreases angiogenesis and proliferation in vivo, indicating that regulation of the AKT pathway may play an important therapeutic role [33].

Mitogen-inducible gene 6 (MIG-6) functions to suppress endometrial cancer in the human and mouse uterus [34, 35]. *Mig-6* is an important mediator of P4 signaling in that it inhibits E2-mediated epithelial proliferation in the uterus [35, 36]. MIG-6 loss is uniquely associated with infertility and endometrial cancer [35, 37–39], but the effects of MIG-6 loss have not been specifically investigated in regulation of epithelial proliferation of endometrial cancer. In this study, we demonstrate that *Mig-6* is pivotal in the suppression of epithelial proliferation through its inhibition of AKT activation. Specifically, we show that P4 inhibition of endometrial tumorigenesis is mediated by MIG-6 inhibition of AKT phosphorylation.

## Methods

### Animals and treatments

Mice were maintained for and used in the designated animal care facility according to the Michigan State University institutional guidelines. All animal procedures were approved by the Institutional Animal Care and Use Committee of Michigan State University. Mice were housed in standard cages (up to 5 animals per cage) in rooms with 12 h light/dark cycle, controlled temperature and humidity under specific pathogen-free conditions. Campus Animal Resources at Michigan State University provides veterinary care, daily husbandry and health checks, procurement, and other administrative support for research in biomedical housing facilities and assists with animal health. Animals are observed daily by animal care staff that have additional training in laboratory animal sciences and species-specific handling and husbandry.

To generate uterine epithelial specific *Mig-6* knockout mice, *Mig-6*^*f/f*^ mice were crossed with *Sprr2f*^*cre/+*^ mice [40]. Control (*Mig-6*^*f/f*^) and endometrial epithelial cell-specific *Mig-6* knockout mice (*Sprr2f*^*cre/+*^*Mig-6*^*f/f*^; *Mig-6* ^*d/d*^) [41] were used to investigate the effect of epithelial *Mig-6* ablation on the uterus.

Vehicle (beeswax) or P4 (40 mg/pellet) pellets were placed subcutaneously into control (*Mig-6*^*f/f*^) and *Sprr2f*^*cre+*^*Mig-6*^*f/f*^ mice respectively at 10 weeks of age for 1 week (*n* = 6/treatment/genotype). To avoid any possibility of pain and/or distress to the animal, all surgical procedures were performed under anesthesia. Mice were anesthetized with isoflurane (3% isoflurane in oxygen by inhalation). All surgeries were conducted in dedicated surgical suites using aseptic procedures. Recuperating animals, under close supervision, were kept warm until full postoperative recovery is achieved. Animals were under anesthetic for a maximum of 20 min, and recovery from surgery normally occurs within 30 min as evidence by sternal recumbency, followed by normal ambulation, grooming and feeding. If discomfort is observed, the animals were provided Ketoprofen at a dose of 5 mg/kg as an analgesic. At the end of a given study, all mice were humanely euthanized by cervical dislocation under isoflurane anesthesia or by carbon dioxide asphyxiation and then the uteri from control and *Sprr2f*^*cre+*^*Mig-6*^*f/f*^ mice were collected to investigate the effect of P4 on the development of endometrial hyperplasia.

### Immunohistochemistry and analysis

Immunohistochemistry analysis was performed as previously described [41]. Briefly, uterine sections were pre-incubated with 10% normal goat serum in PBS prior to exposure to anti-PGR (SC-538; Santa Cruz Biotechnology, Dallas, TX), anti-ESR1 (SC-543; Santa Cruz Biotechnology, Dallas, TX), anti-AKT (CS-4691; Cell Signaling, Danvers, MA), anti-pAKT (CS-4060; Cell Signaling, Danvers, MA), and anti-Ki67 (BD5506090; BD Biosciences, San Jose, CA) as appropriate primary antibodies. Positive signaling was detected with the DAB Peroxidase Substrate Kit (SK-4100; Vector Laboratories, Burlingame, CA). The H-score was calculated as previously reported [42]. The overall H-score ranged from 0 to 300.

### Cell culture and transient transfection

Ishikawa (99,040,201; Sigma–Aldrich, St. Louis, MO) and HEC1A (HTB-112; ATCC, Manassas, VA) Cell lines are maintained in Dulbecco’s modified Eagle’s medium/Nutrient Mixture F-12 (DMEM/F12; Gibco BRL, Gaithersburg, MD) with 10% (*v*/v) fetal bovine serum (FBS; Gibco BRL, Gaithersburg, MD), and 1% (v/v) penicillin streptomycin (P/S; Gibco BRL, Gaithersburg, MD) at 37 °C under 5% CO_2_. FLAG-tagging MIG-6 expression vectors were transfected using Lipofectamine 2000 reagent (Invitrogen Crop., Carlsbad, CA) according to the manufacturer’s instructions.

### Immunoprecipitation

Immunoprecipitation was performed as described previously [38]. Briefly, Ishikawa and HEC1A cells were transfected with the FLAG-MIG-6 expression vectors. Immunoprecipitation was performed with Flag antibody (F1804; Sigma–Aldrich, St. Louis, MO). Protein interactions were examined by Western blot analysis.

### Western blot analysis

Western blot analysis was performed as previously described [41]. Membrane was blocked with Casein (0.5% *v*/v) prior to exposure to anti-AKT (CS-4691; Cell Signaling, Danvers, MA), anti-pAKT (CS-4060; Cell Signaling, Danvers, MA), and anti-Flag (F1804; Sigma-Aldrich, St. Louis, MO) antibodies. Anti-actin (SC-1615, Santa Cruz Biotechnology, Dallas, TX) was used for loading control.

### Statistical analysis

For data with only two groups, Student’s t-test was used. For data containing more than two groups, an analysis of variance (ANOVA) test was used, followed by Tukey or Bonferroni test for pairwise t-tests. All statistical analyses were performed using the Instat package from GraphPad (San Diego, CA, USA).

## Results

### A decrease of stromal PGR and ESR1 expression in *Sprr2f*^*cre+*^*Mig-6*^*f/f*^ mice

**Fig. 1:**
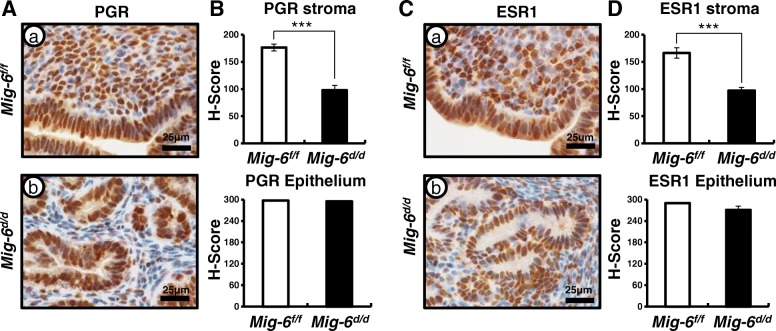
A decrease of stromal PGR and ESR1 expression in *Mig-6*^*d/d*^ mice. Immunohistochemical analysis for PGR (**A**) and ESR1 (**C**) in control (a) and *Mig-6*^*d/d*^ (b) mice. H-score in stroma and epithelial cells for PGR (**B**) and ESR1 (**D**). The results represent the mean ± SEM. ***, *p* < 0.001

Previously, we reported that the hyperplastic phenotype of endometrial epithelial cell specific *Mig-6* knockout (*Sprr2f*^*cre+*^*Mig-6*^*f/f*^; *Mig-6*^*d/d*^) mice were observed at 10 weeks of age [43]. Endometrial cancer displays an imbalance in steroid hormone action [14–17]. PGR expression has been shown to be a prognostic factor for endometrial cancer [44–46]. Therefore, we first examined expression of PGR and ESR1 in *Mig-6*^*d/d*^ mice. Immunohistochemical analysis indicated that levels of PGR and ESR1 were significantly decreased in the stromal cells of *Mig-6*^*d/d*^ mice compared to control (*Mig-6*^*f/f*^) mice at 10 weeks of age (*n* = 6/genotype). However, the expression of PGR and ESR1 in the epithelium were not changed in the uteri of *Mig-6*^*d/d*^ mice as compared to control (Fig. 1). These data suggest that dysregulation of PGR and ESR1 expression in the stroma may play an important role for the development of endometrial hyperplasia.

### Aberrant activation of AKT signaling in *Sprr2f*^*cre+*^*Mig-6*^*f/f*^ mice

**Fig. 2:**
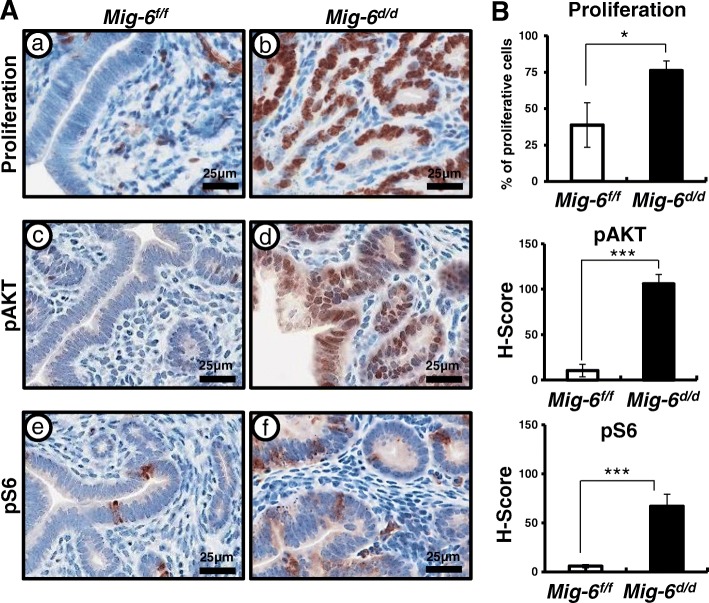
Aberrant activation of proliferation and AKT signaling in *Mig-6*^*d/d*^ mice. (**A**) The expression of Ki67, pAKT, and pS6 in the uteri of *Mig-6*^*f/f*^ (a,c,e) and *Mig-6*^*d/d*^ (b, d, and f) mice. (**B**) Quantification of Ki67 positive cells and H-score in epithelial cells of *Mig-6*^*f/f*^ and *Mig-6*^*d/d*^ mice. The results represent the mean ± SEM. *, *p* < 0.05; ***, *p* < 0.001

AKT is frequently hyperactivated in human cancers [47]. To determine if the observed hyperplastic phenotype was due to activated AKT signaling, we examined the expression of total AKT, phospho-AKT (pAKT), and phospho-S6 (pS6), a downstream marker of active AKT signaling in the uteri of control and *Mig-6*^*d/d*^ mice. First, we examined cell proliferation by Ki67 staining (*n* = 6/genotype). The IHC results revealed a significant increase of uterine epithelial proliferation in *Mig-6*^*d/d*^ mice (Fig. 2a-b). Interestingly, we found that pAKT and pS6 were highly elevated in the epithelial cells of *Mig-6*^*d/d*^ mice at 10 weeks of age as compared to control mice (Fig. 2). However, total AKT levels were not changed among the genotypes (Additional file 1: Figure S1). These data suggest that MIG-6 suppresses endometrial epithelial proliferation via inhibition of AKT phosphorylation.

### The effect of P4 treatment on the development of endometrial hyperplasia

**Fig. 3:**
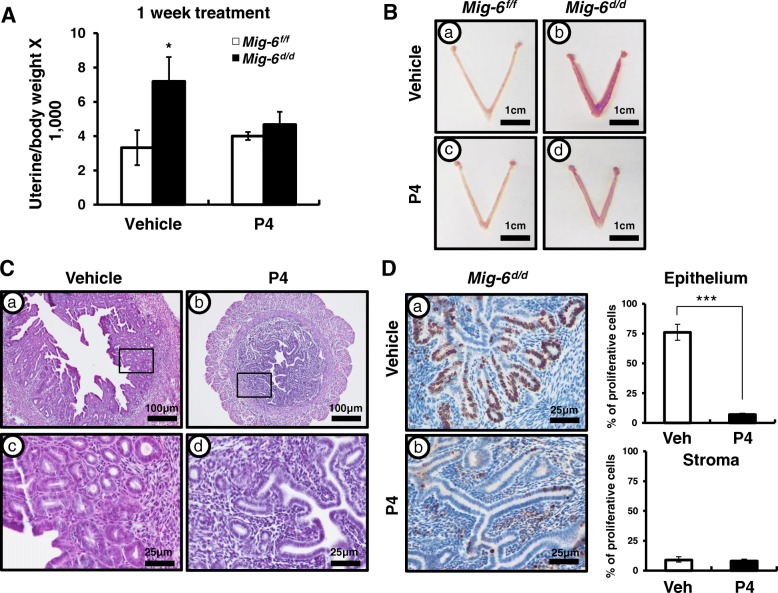
Effects of P4 on *Mig-6*^*f/f*^ and *Mig-6*^*d/d*^ mice. (**A**) Uterine/body ratio were significantly decreased in *Mig-6*^*d/d*^ mice compared to vehicle-treated *Mig-6*^*KO*^ mice after P4 treatment. (**B**) Gross morphology and (**C**) Hematoxylin-eosin staining after vehicle and P4 treatment. (**D**) Immunohistochemical analysis and quantification of Ki67 in *Mig-6*^*f/f*^ and *Mig-6*^*d/d*^ mice. The results represent the mean ± SEM. *, *p* < 0.05; ***, *p* < 0.001

Exposure to P4 is a negative risk factor for endometrial cancer [48]. Additionally, it is well known that endometrial cancer is E2-dependent and that progestin therapy has been successful in slowing the growth of endometrial tumors in women who are poor surgical candidates and premenopausal women with complex atypical hyperplasia and early-stage endometrioid endometrial cancer who had a strong desire to preserve their fertility [22, 23, 49–54]. To assess the effect of P4 treatment on epithelial ablation of *Mig-6*, we placed P4 or vehicle pellets into the control and *Mig-6*^*d/d*^ mice subcutaneously at 10 weeks of age (*n* = 6/treatment/genotype). After 1 week of the P4 treatment, *Mig-6*^*d/d*^ mice exhibited a significantly decreased uterine weight compared to vehicle-treated *Mig-6*^*d/d*^ mice (Fig. 3a and b). Histological analysis showed that the development of uterine hyperplasia was not evident in *Mig-6*^*d/d*^ mice after P4 treatment (Fig. 3c). P4 treatment also led to decreased proliferation in the epithelial cells of *Mig-6*^*d/d*^ mice as compared to vehicle-treated *Mig-6*^*d/d*^ mice (Fig. 3d). These data suggest that the hyperplastic phenotype of *Mig-6*^*d/d*^ mice was responsive to P4 treatment, returning the morphology to normal.

### The recovery of steroid hormone and AKT signaling by P4 treatment in *Sprr2f*^*cre+*^*Mig-6*^*f/f*^ mice

**Fig. 4:**
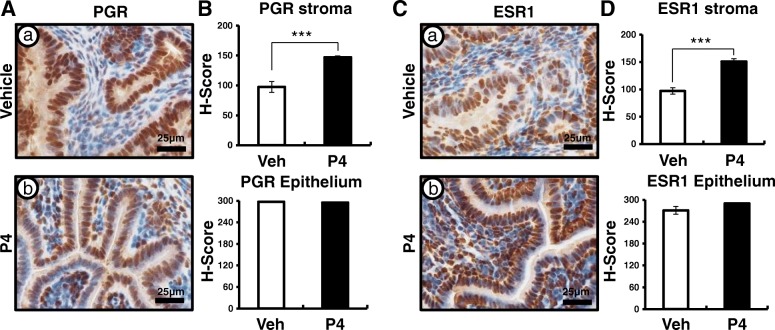
Recovery of stroma PGR and ESR1 expression in *Mig-6*^*d/d*^ mice after P4 treatment. Immunohistochemical analysis for PGR (**A**) and ESR1 (**C**) in vehicle- (a) and *P4-*(b) treated *Mig-6*^*d/d*^ mice. Quantification of PGR (**B**) and ESR1 (**D**) by H-score. The results represent the mean ± SEM. ***, *p* < 0.001

The expression of PGR and ESR1 is strongly correlated with the prognosis of endometrial cancer [55]. Therefore, we examined the expression of PGR and ESR1 using immunohistochemistry (*n* = 6/treatment). The expression of PGR and ESR1 were significantly increased in the stroma of *Mig-6*^*d/d*^ mice after P4 treatment (Fig. 4). These data indicated that P4 treatment activates nuclear receptors signaling at endometrial stromal cells of *Mig-6*^*d/d*^ mice.

**Fig. 5:**
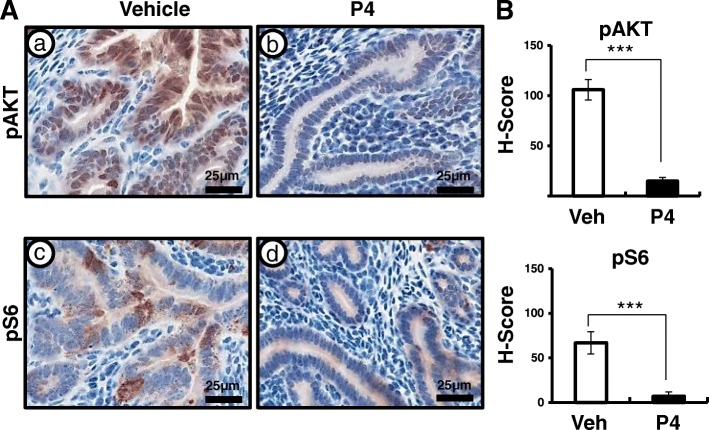
AKT signaling is down-regulated after P4 treatment in *Mig-6*^*d/d*^ mice. (**A**) Immunohistochemical analysis of pAKT and pS6 in vehicle and P4-treated *Mig-6*^*d/d*^ mice. (**B**) Quantification of pAKT and pS6 positive cells in epithelial cells of *Mig-6*^*f/f*^ and *Mig-6*^*d/d*^ mice after P4 treatment. The results represent the mean ± SEM. ***, *p* < 0.001

Next, we examined the expression of total AKT, pAKT, and pS6 using immunohistochemistry in the uteri of control and *Mig-6*^*d/d*^ mice after P4 treatment to investigate whether the suppression of hyperplastic phenotype observed was due to recovered AKT signaling. Total AKT levels were not changed after P4 treatment (Additional file 2: Figure S2). However, aberrant activation of AKT signaling was significantly decreased in the uteri of P4-treated *Mig-6*^*d/d*^ mice as compared to vehicle-treated *Mig-6*^*d/d*^ mice (Fig. 5). These data suggest that P4 treatment suppresses aberrant activation of AKT signaling in endometrial hyperplasia of *Mig-6*^*d/d*^ mice.

### MIG-6 regulates AKT phosphorylation dose-dependently and interacts with AKT

**Fig. 6:**
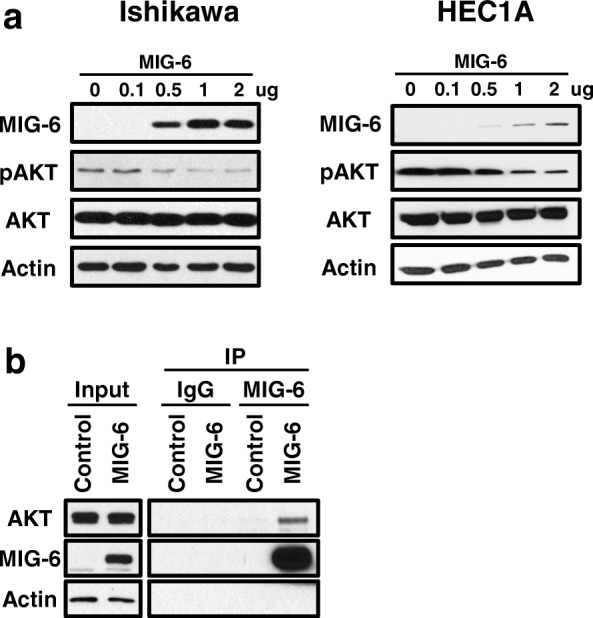
AKT phosphorylation were regulated by MIG-6 expression dose-dependently (**a**) Western Blot analysis of MIG-6, pAKT, AKT, and Actin in FLAG-MIG-6 transfected Ishikawa and HEC1A cells. Actin was used as sample-loading control. (**b**) The interaction between MIG-6 and AKT by immunoprecipitation

In order to examine effects of MIG-6 on AKT, we performed experiments on endometrial cancer cell lines, Ishikawa and HEC1A cells. We transfected to Ishikawa and HEC1A cells dose-dependently with FLAG-tagged MIG-6 (FLAG-MIG-6). Following MIG-6 introduction we examined levels of AKT and pAKT at 24-h. The levels of AKT phosphorylation were highly decreased by FLAG-MIG-6 in a dose dependent manner whereas AKT levels were unchanged (Fig. 6a). We next examined whether MIG-6 physically interacts with AKT. Ishikawa cells were transfected with FLAG-MIG-6, and the lysates were immunoprecipitated with FLAG antibody. FLAG immunoprecipitates were then probed with AKT and MIG-6 specific antibodies, indicating that MIG-6 physically interacts with AKT (Fig. 6b). These results suggest that MIG-6 inhibits AKT phosphorylation through a protein-protein interaction, highlighting its important role in the regulation of epithelial proliferation.

## Discussion

In this study, we evaluated whether MIG-6 suppresses endometrial epithelial proliferation via inhibition of AKT phosphorylation. P4 plays an inhibitory role on E2 stimulated proliferation of uterine epithelial cells [56]. Disruption of steroidal control results in unopposed E2, leading to endometrial cancer [17]. *Mig-6* is a target of P4 and PGR, and its deletion in the uterus leads to enhanced epithelial proliferation [35]. The majority of endometrial cancers exhibit actively proliferating epithelial cells and increased AKT signaling [57–59]. The Cancer Genome Atlas analysis demonstrated an increased AKT activity in endometrioid endometrial tumors [25]. Activated AKT signaling enhances cell proliferation as well as cell survival through the inhibition of proapoptotic proteins [27]. Expression of PGR (PR-A and PR-B) and ESRs (ESR1 and ESR2) has been reported as prognostic factors for endometrial carcinoma [44–46]. We evaluated that stromal PGR and ESR1 expression was significantly decreased in the uteri of *Mig-6*^*d/d*^ when compared to control mice (Fig. 1). We showed elevated phosphorylation of AKT resulting in enhanced epithelial proliferation (Fig. 2). Stromal PGR and P4 signaling is necessary and sufficient to mediate the antiproliferative effects of P4 on E2-induced epithelial cell proliferation [60, 61]. However, activation of AKT reduces PR-B transcriptional activity in Ishikawa cells and *Pten*^*d/d*^ conditional mouse model of endometrioid endometrial cancer [33]. AKT also reduces PGR expression levels in breast cancer cells, endometrial cancer cells, and uterine stromal cells affected by endometriosis [62–64]. However, exactly how signaling occurs between AKT and P4 resistance in endometrial epithelial and stromal interaction is unclear. Filling this knowledge gap is critical to understanding P4 resistance.

P4 resistance is defined by the decreased responsiveness to bioavailable P4 of target tissue [65]. Lack of P4 activity contributes significantly to uterine pathophysiology. P4 resistance is now considered a central element in women’s diseases such as infertility, endometriosis, and endometrial cancer [66–69], but the mechanism of P4 resistance in women’s diseases remains unknown. We have demonstrated that *Mig-6*^*d/d*^ mice exhibiting normal P4 responses and P4 treatment for 1 week is sufficient to restore endometrial hyperplasia to normal (Fig. 3). We treated the mice in the beginning of endometrial hyperplasia and the data suggest P4 treatment at an early time point can be one of the reasons to reverse endometrial hyperplasia to normal. Therefore, further study on the effects of P4 treatment on endometrial turmorigenesis associated with its development and progression are required.

Determining the molecular mechanisms by which steroid hormones control the physiology of the uterus is of utmost importance to understanding and overcoming P4 resistance. However, resistance to P4 treatment has led to limiting the use of P4 therapy in endometrial cancer due to its low response rates [19–23]. The optimal method for treating and surveilling patients with conservatively treated endometrial cancer is not known. Therefore, the identification of the molecular pathways that link P4 resistance to endometrial cancer development can potentially provide novel targets for the prevention or treatment of this malignancy. We showed that AKT signaling is down-regulated after P4 treatment in *Mig-6*^*d/d*^ mice (Fig. 5). These data suggest that treatment with an AKT inhibitor could be a viable alternative for overcoming the P4-resistant endometrial hyperplasia and cancer.

We found that MIG-6 decreased AKT phosphorylation in Ishikawa and HEC1A cell lines in a dose-dependent manner. Immunoprecipitation showed that there is protein interaction between MIG-6 and AKT, suggesting that MIG-6 suppresses E2-induced epithelial cell proliferation through AKT interactions (Fig. 6). However, the exact molecular mechanism by which interaction regulates the phosphorylation of AKT is not clear. Further studies will be required to determine exact molecular mechanism.

We have shown the prevention effect of P4 with *Mig-6*^*d/d*^ mice [43]. We treated *Mig-6*^*d/d*^ mice with P4 before developing endometrial hyperplasia and found that P4 prevented the development of endometrial hyperplasia by inhibiting epithelial STAT3 phosphorylation, resulting in a decrease of epithelial proliferation. The molecular mechanisms in the regulation of epithelial proliferation by AKT and STAT3 as well as steroid hormone signaling remains to be further studied during endometrial tumorigenesis. Our data support that the activation of stromal signaling by P4 treatment can contribute to the development of endometrial hyperplasia and the cross-talk between AKT/STAT3 and PGR/ESR1 is critical to inhibit the endometrial hyperplasia.

## Conclusions

Overall, our study suggests that the negative regulation of AKT phosphorylation by activated stroma signaling including *Mig-6* has an important role in the regulation of epithelial cell proliferation during endometrial hyperplasia development and progression. Our results contribute to the understanding of the etiological and molecular mechanisms of epithelial cell proliferation and to the development of new therapeutic approaches for treating endometrial hyperplasia and cancer.

## Additional files

Additional file 1: **Figure S1** Total AKT level is not changed in *Mig-6*^*f/f*^ and *Mig-6*^*d/d*^ mice. (A) The expression of AKT in the uteri of *Mig-6*^*f/f*^ (a) and *Mig-6*^*d/d*^ (b) mice and (B) H-score of AKT in the uteri of *Mig-6*^*f/f*^ and *Mig-6*^*d/d*^ mice. (PPTX 251 kb)

Additional file 2: **Figure S2** Total AKT level is not changed in *Mig-6*^*d/d*^ mice after P4 treatment. (A) The expression of AKT in the uteri of vehicle (a) and P4 (b) treated *Mig-6*^*d/d*^ mice and (B) H-score of AKT in the uteri of vehicle and P4 treated *Mig-6*^*d/d*^ mice. (PPTX 407 kb)

## Abbreviations

ESR1: Estrogen receptor α
ESR2: Estrogen receptor β
PGR: Progesterone receptor
PI3K: Phosphoinositide 3-kinase
PTEN: Phosphatase and tensin homolog
S6: Ribosomal protein S6 kinase

## References

[1] Siegel RL, Miller KD, Jemal A (2016). Cancer statistics, 2016. CA Cancer J Clin.

[2] Byun JM, Jeong DH, Kim YN, Cho EB, Cha JE, Sung MS, Lee KB, Kim KT (2015). Endometrial cancer arising from atypical complex hyperplasia: the significance in an endometrial biopsy and a diagnostic challenge. Obstet Gynecol Sci.

[3] Saso S, Chatterjee J, Georgiou E, Ditri AM, Smith JR, Ghaem-Maghami S (2011). Endometrial cancer. BMJ.

[4] Sherman ME (2000). Theories of endometrial carcinogenesis: a multidisciplinary approach. Mod Pathol.

[5] ACOG Committee Opinion No. 444 (2009). choosing the route of hysterectomy for benign disease. Obstet Gynecol.

[6] Temkin SM, Minasian L, Noone AM (2016). The end of the hysterectomy epidemic and endometrial Cancer incidence: what are the unintended consequences of declining hysterectomy rates?. Front Oncol.

[7] Pellerin GP, Finan MA (2005). Endometrial cancer in women 45 years of age or younger: a clinicopathological analysis. Am J Obstet Gynecol.

[8] Chassot PG, Delabays A, Spahn DR (2002). Preoperative evaluation of patients with, or at risk of, coronary artery disease undergoing non-cardiac surgery. Br J Anaesth.

[9] Charytan DM, Li S, Liu J, Herzog CA (2012). Risks of death and end-stage renal disease after surgical compared with percutaneous coronary revascularization in elderly patients with chronic kidney disease. Circulation.

[10] Varon J, Marik PE (2008). Perioperative hypertension management. Vasc Health Risk Manag.

[11] Jareid M, Thalabard JC, Aarflot M, Bovelstad HM, Lund E, Braaten T. Levonorgestrel-releasing intrauterine system use is associated with a decreased risk of ovarian and endometrial cancer, without increased risk of breast cancer. Results from the NOWAC study. Gynecol Oncol. 2018;149(1):127–32. 10.1016/j.ygyno.2018.02.006 29482839

[12] Fan Z, Li H, Hu R, Liu Y, Liu X, Gu L (2018). Fertility-preserving treatment in young women with grade 1 presumed stage IA endometrial adenocarcinoma: a meta-analysis. Int J Gynecol Cancer.

[13] Pal N, Broaddus RR, Urbauer DL, Balakrishnan N, Milbourne A, Schmeler KM, Meyer LA, Soliman PT, Lu KH, Ramirez PT (2018). Treatment of low-risk endometrial Cancer and complex atypical hyperplasia with the Levonorgestrel-releasing intrauterine device. Obstet Gynecol.

[14] Bokhman JV (1983). Two pathogenetic types of endometrial carcinoma. Gynecol Oncol.

[15] Sherman ME, Sturgeon S, Brinton LA, Potischman N, Kurman RJ, Berman ML, Mortel R, Twiggs LB, Barrett RJ, Wilbanks GD (1997). Risk factors and hormone levels in patients with serous and endometrioid uterine carcinomas. Mod Pathol.

[16] Deligdisch L, Holinka CF (1987). Endometrial carcinoma: two diseases?. Cancer Detect Prev.

[17] Kurman RJ, Kaminski PF, Norris HJ (1985). The behavior of endometrial hyperplasia. A long-term study of "untreated" hyperplasia in 170 patients. Cancer.

[18] Jick SS (1993). Combined estrogen and progesterone use and endometrial cancer. Epidemiology.

[19] Ramirez PT, Frumovitz M, Bodurka DC, Sun CC, Levenback C (2004). Hormonal therapy for the management of grade 1 endometrial adenocarcinoma: a literature review. Gynecol Oncol.

[20] Park H, Seok JM, Yoon BS, Seong SJ, Kim JY, Shim JY, Park CT (2012). Effectiveness of high-dose progestin and long-term outcomes in young women with early-stage, well-differentiated endometrioid adenocarcinoma of uterine endometrium. Arch Gynecol Obstet.

[21] Decruze SB, Green JA (2007). Hormone therapy in advanced and recurrent endometrial cancer: a systematic review. Int J Gynecol Cancer.

[22] Randall TC, Kurman RJ (1997). Progestin treatment of atypical hyperplasia and well-differentiated carcinoma of the endometrium in women under age 40. Obstet Gynecol.

[23] Kim YB, Holschneider CH, Ghosh K, Nieberg RK, Montz FJ (1997). Progestin alone as primary treatment of endometrial carcinoma in premenopausal women. Report of seven cases and review of the literature. Cancer.

[24] Mittal N, Malpani S, Dyson M, Ono M, Coon JS, Kim JJ, Schink JC, Bulun SE, Pavone ME (2014). Fenretinide: a novel treatment for endometrial cancer. PLoS One.

[25] Kandoth C, Schultz N, Cherniack AD, Akbani R, Liu Y, Shen H, Robertson AG, Pashtan I, Shen R, Cancer Genome Atlas Research N (2013). integrated genomic characterization of endometrial carcinoma. Nature.

[26] Chambliss KL, Yuhanna IS, Anderson RG, Mendelsohn ME, Shaul PW (2002). ERbeta has nongenomic action in caveolae. Mol Endocrinol.

[27] Engelman JA, Luo J, Cantley LC (2006). The evolution of phosphatidylinositol 3-kinases as regulators of growth and metabolism. Nat Rev Genet.

[28] Cantley LC (2004). The role of phosphoinositide 3-kinase in human disease. Harvey Lect.

[29] Manning BD, Toker A (2017). AKT/PKB signaling: navigating the network. Cell.

[30] Carnero A (2010). The PKB/AKT pathway in cancer. Curr Pharm Des.

[31] Brazil DP, Yang ZZ, Hemmings BA (2004). Advances in protein kinase B signalling: AKTion on multiple fronts. Trends Biochem Sci.

[32] Terakawa N, Kanamori Y, Yoshida S (2003). Loss of PTEN expression followed by Akt phosphorylation is a poor prognostic factor for patients with endometrial cancer. Endocr Relat Cancer.

[33] Lee II, Maniar K, Lydon JP, Kim JJ (2016). Akt regulates progesterone receptor B-dependent transcription and angiogenesis in endometrial cancer cells. Oncogene.

[34] Kim TH, Lee DK, Franco HL, Lydon JP, Jeong JW (2010). ERBB receptor feedback inhibitor 1 regulation of estrogen receptor activity is critical for uterine implantation in mice. Biol Reprod.

[35] Jeong JW, Lee HS, Lee KY, White LD, Broaddus RR, Zhang YW, Vande Woude GF, Giudice LC, Young SL, Lessey BA (2009). Mig-6 modulates uterine steroid hormone responsiveness and exhibits altered expression in endometrial disease. Proc Natl Acad Sci U S A.

[36] Jeong JW, Lee KY, Kwak I, White LD, Hilsenbeck SG, Lydon JP, DeMayo FJ (2005). Identification of murine uterine genes regulated in a ligand-dependent manner by the progesterone receptor. Endocrinology.

[37] Kim TH, Franco HL, Jung SY, Qin J, Broaddus RR, Lydon JP, Jeong JW (2010). The synergistic effect of Mig-6 and Pten ablation on endometrial cancer development and progression. Oncogene.

[38] Kim TH, Lee DK, Cho SN, Orvis GD, Behringer RR, Lydon JP, Ku BJ, McCampbell AS, Broaddus RR, Jeong JW (2013). Critical tumor suppressor function mediated by epithelial Mig-6 in endometrial cancer. Cancer Res.

[39] Kim TH, Yoo JY, Kim HI, Gilbert J, Ku BJ, Li J, Mills GB, Broaddus RR, Lydon JP, Lim JM (2014). Mig-6 suppresses endometrial cancer associated with Pten deficiency and ERK activation. Cancer Res.

[40] Contreras CM, Akbay EA, Gallardo TD, Haynie JM, Sharma S, Tagao O, Bardeesy N, Takahashi M, Settleman J, Wong KK (2010). Lkb1 inactivation is sufficient to drive endometrial cancers that are aggressive yet highly responsive to mTOR inhibitor monotherapy. Dis Model Mech.

[41] Kim BG, Yoo JY, Kim TH, Shin JH, Langenheim JF, Ferguson SD, Fazleabas AT, Young SL, Lessey BA, Jeong JW (2015). Aberrant activation of signal transducer and activator of transcription-3 (STAT3) signaling in endometriosis. Hum Reprod.

[42] Ishibashi H, Suzuki T, Suzuki S, Moriya T, Kaneko C, Takizawa T, Sunamori M, Handa M, Kondo T, Sasano H (2003). Sex steroid hormone receptors in human thymoma. J Clin Endocrinol Metab.

[43] Yoo JY, Yang WS, Lee JH, Kim BG, Broaddus RR, Lim JM, Kim TH, Jeong JW. MIG-6 negatively regulates STAT3 phosphorylation in uterine epithelial cells. Oncogene. 2017;37:255–62. 10.1038/onc.2017.335 PMC5764811 28925396

[44] Kleine W, Maier T, Geyer H, Pfleiderer A (1990). Estrogen and progesterone receptors in endometrial cancer and their prognostic relevance. Gynecol Oncol.

[45] Nyholm HC, Nielsen AL, Lyndrup J, Dreisler A, Thorpe SM (1993). Estrogen and progesterone receptors in endometrial carcinoma: comparison of immunohistochemical and biochemical analysis. Int J Gynecol Pathol.

[46] Fukuda K, Mori M, Uchiyama M, Iwai K, Iwasaka T, Sugimori H (1998). Prognostic significance of progesterone receptor immunohistochemistry in endometrial carcinoma. Gynecol Oncol.

[47] Sansal I, Sellers WR (2004). The biology and clinical relevance of the PTEN tumor suppressor pathway. J Clin Oncol.

[48] Grosskinsky CM, Halme J (1993). Endometriosis: the host response. Baillieres Clin Obstet Gynaecol.

[49] Kaku T, Yoshikawa H, Tsuda H, Sakamoto A, Fukunaga M, Kuwabara Y, Hataeg M, Kodama S, Kuzuya K, Sato S (2001). Conservative therapy for adenocarcinoma and atypical endometrial hyperplasia of the endometrium in young women: central pathologic review and treatment outcome. Cancer Lett.

[50] Ogawa S, Koike T, Shibahara H, Ohwada M, Suzuki M, Araki S, Sato I (2001). Assisted reproductive technologies in conjunction with conservatively treated endometrial adenocarcinoma. A case report. Gynecol Obstet Investig.

[51] Mitsushita J, Toki T, Kato K, Fujii S, Konishi I (2000). Endometrial carcinoma remaining after term pregnancy following conservative treatment with medroxyprogesterone acetate. Gynecol Oncol.

[52] Gallos ID, Ganesan R, Gupta JK (2013). Prediction of regression and relapse of endometrial hyperplasia with conservative therapy. Obstet Gynecol.

[53] Bovicelli A, D'Andrilli G, Giordano A, De Iaco P (2013). Conservative treatment of early endometrial cancer. J Cell Physiol.

[54] Koskas M, Azria E, Walker F, Luton D, Madelenat P, Yazbeck C (2012). Progestin treatment of atypical hyperplasia and well-differentiated adenocarcinoma of the endometrium to preserve fertility. Anticancer Res.

[55] Supernat A, Lapinska-Szumczyk S, Majewska H, Gulczynski J, Biernat W, Wydra D, Zaczek AJ (2014). Tumor heterogeneity at protein level as an independent prognostic factor in endometrial cancer. Transl Oncol.

[56] Martin L, Finn CA, Trinder G (1973). Hypertrophy and hyperplasia in the mouse uterus after oestrogen treatment: an autoradiographic study. J Endocrinol.

[57] Sivridis E, Giatromanolaki A (2004). Endometrial adenocarcinoma: beliefs and scepticism. Int J Surg Pathol.

[58] Ejskjaer K, Sorensen BS, Poulsen SS, Forman A, Nexo E, Mogensen O (2007). Expression of the epidermal growth factor system in endometrioid endometrial cancer. Gynecol Oncol.

[59] Khalifa MA, Mannel RS, Haraway SD, Walker J, Min KW (1994). Expression of EGFR, HER-2/neu, P53, and PCNA in endometrioid, serous papillary, and clear cell endometrial adenocarcinomas. Gynecol Oncol.

[60] Janzen DM, Rosales MA, Paik DY, Lee DS, Smith DA, Witte ON, Iruela-Arispe ML, Memarzadeh S (2013). Progesterone receptor signaling in the microenvironment of endometrial cancer influences its response to hormonal therapy. Cancer Res.

[61] Kurita T, Young P, Brody JR, Lydon JP, O'Malley BW, Cunha GR (1998). Stromal progesterone receptors mediate the inhibitory effects of progesterone on estrogen-induced uterine epithelial cell deoxyribonucleic acid synthesis. Endocrinology.

[62] Pant A, Lee II, Lu Z, Rueda BR, Schink J, Kim JJ (2012). Inhibition of AKT with the orally active allosteric AKT inhibitor, MK-2206, sensitizes endometrial cancer cells to progestin. PLoS One.

[63] Cui X, Zhang P, Deng W, Oesterreich S, Lu Y, Mills GB, Lee AV (2003). Insulin-like growth factor-I inhibits progesterone receptor expression in breast cancer cells via the phosphatidylinositol 3-kinase/Akt/mammalian target of rapamycin pathway: progesterone receptor as a potential indicator of growth factor activity in breast cancer. Mol Endocrinol.

[64] Eaton JL, Unno K, Caraveo M, Lu Z, Kim JJ (2013). Increased AKT or MEK1/2 activity influences progesterone receptor levels and localization in endometriosis. J Clin Endocrinol Metab.

[65] Chrousos GP, MacLusky NJ, Brandon DD, Tomita M, Renquist DM, Loriaux DL, Lipsett MB (1986). Progesterone resistance. Adv Exp Med Biol.

[66] Al-Sabbagh M, Lam EW, Brosens JJ (2012). Mechanisms of endometrial progesterone resistance. Mol Cell Endocrinol.

[67] Burney RO, Talbi S, Hamilton AE, Vo KC, Nyegaard M, Nezhat CR, Lessey BA, Giudice LC (2007). Gene expression analysis of endometrium reveals progesterone resistance and candidate susceptibility genes in women with endometriosis. Endocrinology.

[68] Soyal SM, Mukherjee A, Lee KY, Li J, Li H, DeMayo FJ, Lydon JP (2005). Cre-mediated recombination in cell lineages that express the progesterone receptor. Genesis.

[69] Attia GR, Zeitoun K, Edwards D, Johns A, Carr BR, Bulun SE (2000). Progesterone receptor isoform a but not B is expressed in endometriosis. J Clin Endocrinol Metab.

