# Supplementary material for: MIG-6 suppresses endometrial epithelial cell proliferation by inhibiting phospho-AKT
Source: BMC Cancer. 2018 May 29;18:605. doi: 10.1186/s12885-018-4502-7 (PMC5975686; doi:10.1186/s12885-018-4502-7)
Supplement: Supplementary file 1 — Figure S1 Total AKT level is not changed in Mig-6f/f and Mig-6d/d mice. (A) The expression of AKT in the uteri of Mig-6f/f (a) and Mig-6d/d (b) mice and (B) H-score of AKT in the uteri of Mig-6f/f and Mig-6d/d mice. (PPTX 251 kb) [file 12885_2018_4502_MOESM1_ESM.pptx]

## Slide 1
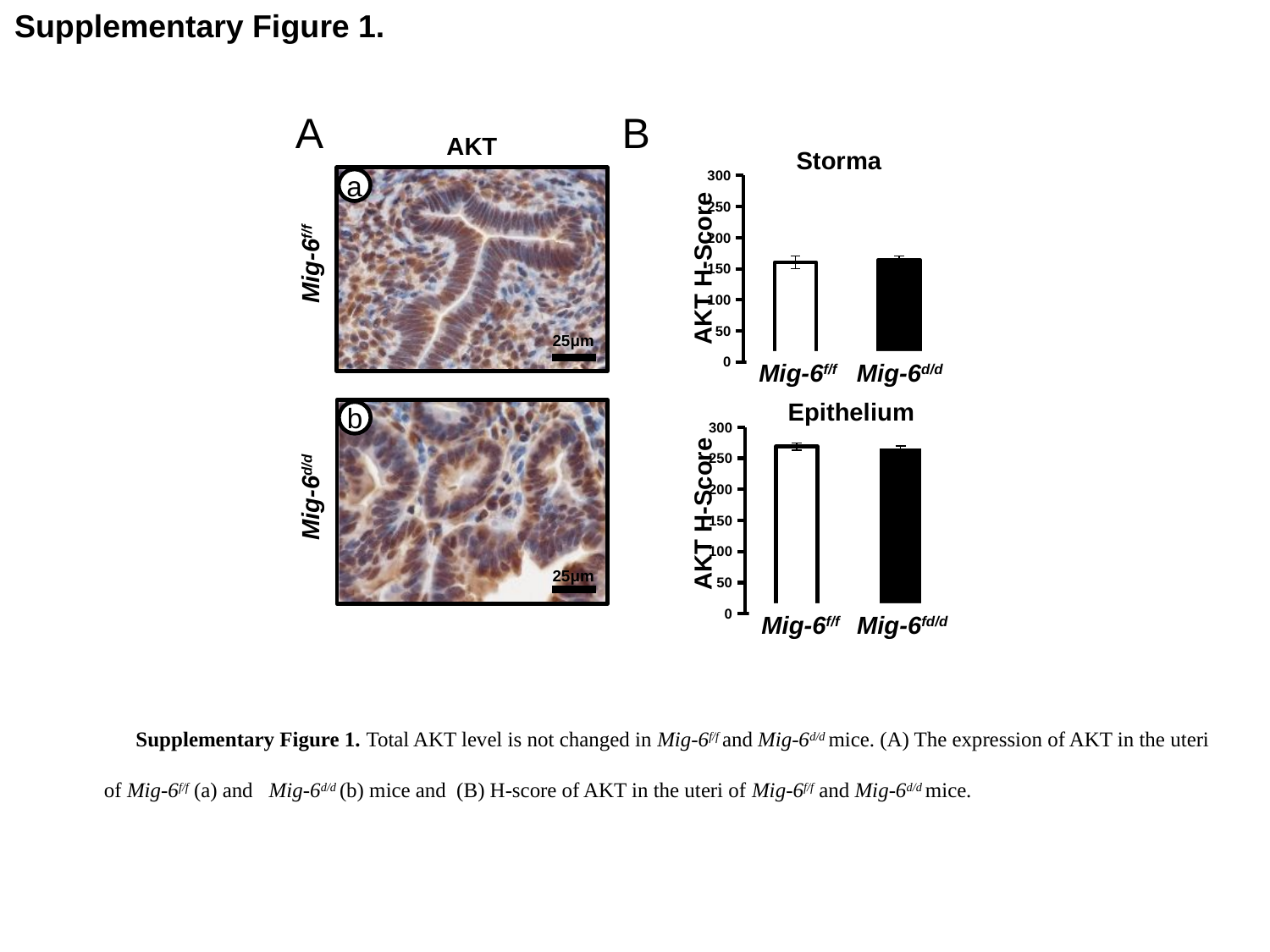

Supplementary Figure 1.
A
B
AKT
Storma
### Chart
| Category | |
|---|---|AKT H-Score
Mig-6f/f
Mig-6d/d
a
Mig-6f/f
25μm
Epithelium
### Chart
| Category | |
|---|---|AKT H-Score
Mig-6f/f
Mig-6fd/d
b
Mig-6d/d
25μm
Supplementary Figure 1. Total AKT level is not changed in Mig-6f/f and Mig-6d/d mice. (A) The expression of AKT in the uteri of Mig-6f/f (a) and Mig-6d/d (b) mice and (B) H-score of AKT in the uteri of Mig-6f/f and Mig-6d/d mice.
