# Supplementary material for: MIG-6 suppresses endometrial epithelial cell proliferation by inhibiting phospho-AKT
Source: BMC Cancer. 2018 May 29;18:605. doi: 10.1186/s12885-018-4502-7 (PMC5975686; doi:10.1186/s12885-018-4502-7)
Supplement: Supplementary file 2 — Figure S2 Total AKT level is not changed in Mig-6d/d mice after P4 treatment. (A) The expression of AKT in the uteri of vehicle (a) and P4 (b) treated Mig-6d/d mice and (B) H-score of AKT in the uteri of vehicle and P4 treated Mig-6d/d mice. (PPTX 407 kb) [file 12885_2018_4502_MOESM2_ESM.pptx]

## Slide 1
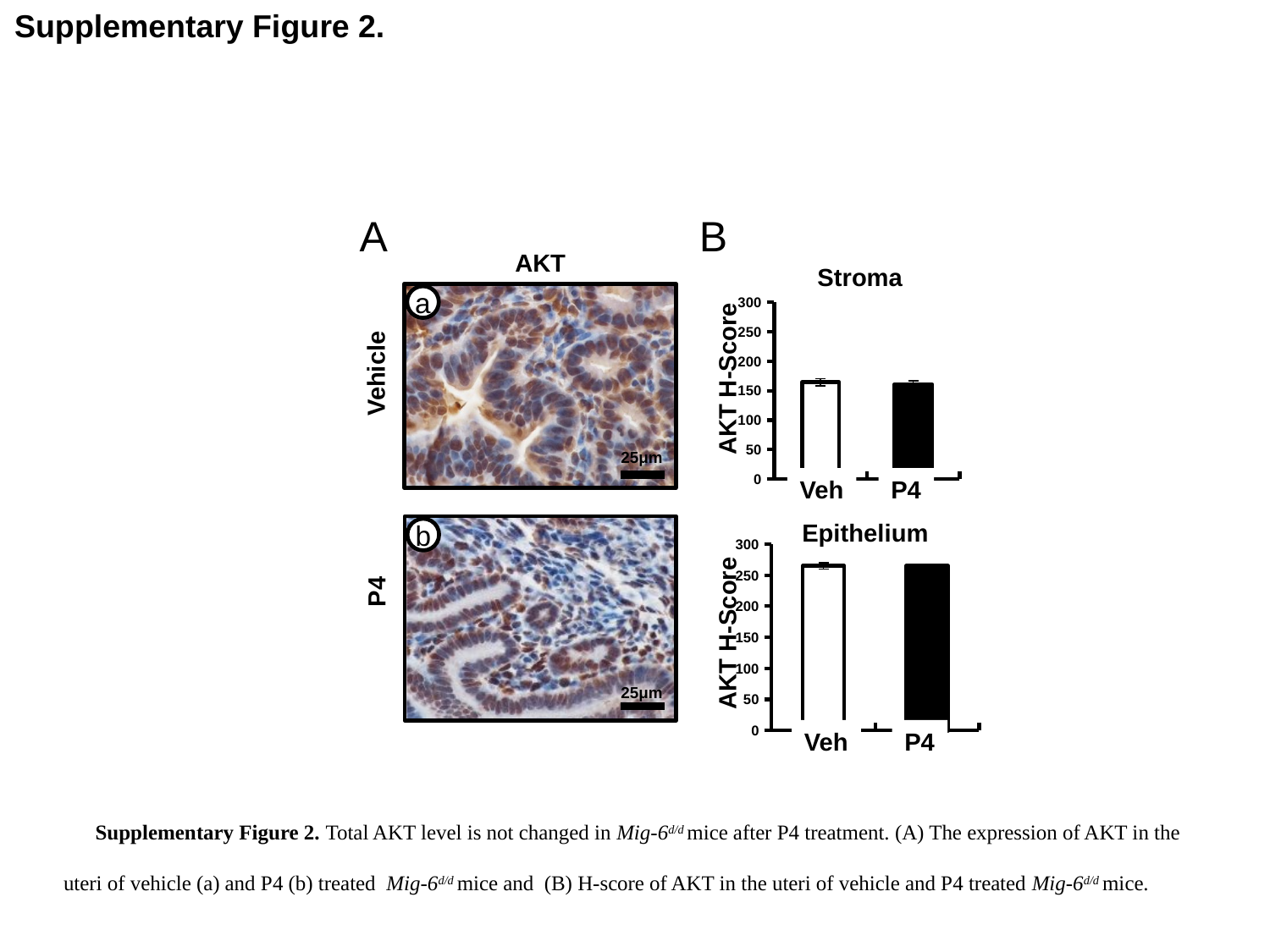

Supplementary Figure 2.
A
B
AKT
Stroma
a
### Chart
| Category | |
|---|---|Vehicle
AKT H-Score
25μm
Veh
P4
Epithelium
b
### Chart
| Category | |
|---|---|P4
AKT H-Score
25μm
Veh
P4
Supplementary Figure 2. Total AKT level is not changed in Mig-6d/d mice after P4 treatment. (A) The expression of AKT in the uteri of vehicle (a) and P4 (b) treated Mig-6d/d mice and (B) H-score of AKT in the uteri of vehicle and P4 treated Mig-6d/d mice.
